# Supplementary material for: Rapid turnover of life-cycle-related genes in the brown algae
Source: Genome Biol. 2019 Feb 14;20:35. doi: 10.1186/s13059-019-1630-6 (PMC6374913; doi:10.1186/s13059-019-1630-6)
Supplement: Supplementary file 3 — Protein alignments, corresponding Gblocks html file and CDS sequences for the lineage conserved gametophyte-biased genes. (ZIP 2109 kb) [file 13059_2019_1630_MOESM3_ESM.zip › Con_GA_alignments/Ec-01_005570.aa_ali.fasta-gb.htm]

Ec-01\_005570.aa.fasta.trans.aa\_ali.fasta


## Gblocks 0.91b Results

Processed file: **Ec-01\_005570.aa.fasta.trans.aa\_ali.fasta**  
Number of sequences: **2**  
Alignment assumed to be: **Protein**  
New number of positions: **421** (selected positions are underlined in blue)

```
                         10        20        30        40        50        60
                 =========+=========+=========+=========+=========+=========+
Ec-01_005570     MWPRRGLMARISLLCTITGGRTPGLR--AMVPLPPSWKSTTTGATTFQMLGNRALKTKPP
MSTRG.22634.1    MWGRRRLMARFSLLCTVAGGRSLPYRGLSSWKQPASSGTSTWIQTSGRGRGCSMLRLGKQ
                 ##########################       ###########################


                         70        80        90       100       110       120
                 =========+=========+=========+=========+=========+=========+
Ec-01_005570     EARGAATTAAPRSAGPAWRKVSHVCSSVVS-APEDVEQSQPQRYESEEQERQPLKHQHQH
MSTRG.22634.1    LQRRGSRGPATAVVSAAWREGIHVRSVVTATAPEDVKHSQSQPLCKLGEQHGELLLRLPS
                 ############################   ###########                  


                        130       140       150       160       170       180
                 =========+=========+=========+=========+=========+=========+
Ec-01_005570     HQQRAGTTTAGGGEGEGRHDSTTRDERLKTTRRAFKP-DTKRMLIIPEEEWPSYPNFSFT
MSTRG.22634.1    GPPDPATTP---GPNQKKHPT---------PSRNFEPGKSKRMLVIPEEEWPRYPKFSFD
                                                         ####################


                        190       200       210       220       230       240
                 =========+=========+=========+=========+=========+=========+
Ec-01_005570     VESECQEGTKARTGTVSTPHGLVQTPAFIFCATKAAIKGLSAETMKECGSQIILSNTYHL
MSTRG.22634.1    IESECREGTSARTATLRTPHGDVQTPAFIFCATKAAIKGLSAETMKECGSQIILSNTYHL
                 ############################################################


                        250       260       270       280       290       300
                 =========+=========+=========+=========+=========+=========+
Ec-01_005570     LVQPGGEMIEKMGGLQKATRWEGPMLTDSGGYQIFSMGHGSVGNEIKGRRGAEGGAEFAP
MSTRG.22634.1    LVQPGGERIEKMGGLQKATRWEGPMLTDSGGYQIFSMGHGSVGDEIKGRRGAEGGAQFSP
                 ############################################################


                        310       320       330       340       350       360
                 =========+=========+=========+=========+=========+=========+
Ec-01_005570     SLVSISEKGARFKSYYDCSIKSLTPERSIEIQRQLGADLVVVLDECTPFHVDKDYTKSSM
MSTRG.22634.1    SLVSISEKGARFKSYHDCSIKSLTPERSIEIQRQLGADLVVVLDECTPFHVDKEYTASSM
                 ############################################################


                        370       380       390       400       410       420
                 =========+=========+=========+=========+=========+=========+
Ec-01_005570     HRTHRWAMRSLREFIDGDDGTQALYGIIQGGVYPDLRNEAVEFVNKAPFFGIAVGGSLGA
MSTRG.22634.1    HRTHRWAMRSLREFIDGDDGAQALYGIVQGGVYPDLREEAVEFVNKTPFFGTAVGGSLGA
                 ############################################################


                        430       440       450       460       470       480
                 =========+=========+=========+=========+=========+=========+
Ec-01_005570     DKATMHQVVSHTMANVRRDRPVHLLGIGGIADIFHGVRQGVDTFDCVHPTRLGRHGGALV
MSTRG.22634.1    DKATMHRVVSQTMAKVRRDRPVHLLGIGGIADIFHGVSQ---SRECVPRKRQS-------
                 #######################################                     


                        490       500       510       520       530       540
                 =========+=========+=========+=========+=========+=========+
Ec-01_005570     KASFWTREQELEEERANQDAAAEAAAAATAEEEASIGGDTMAAGVGASKRKKRRRSGDVR
MSTRG.22634.1    -----AGPQGRRQARCNCLLCSAPVSRPPSPRLCSH-LAPFSLRCGRVLLPHGRLSESPR
                                                              ###############


                        550       560       570       580       590       600
                 =========+=========+=========+=========+=========+=========+
Ec-01_005570     KKRHPVVPR-EHVNLLKGRYREDHRVIDEDCGCPTCRGGYTRAYINHLGRAGELLGGMLV
MSTRG.22634.1    ILQQPLRATPERVWSLEGGFDS----VGGWCRCPSG------------------------
                 #####                                                       


                        610       620       630       640       650       660
                 =========+=========+=========+=========+=========+=========+
Ec-01_005570     SQHNVFFMNELMTSIRTAIAEGRLAEEEDKWLAPGLRARDFHKRAAAEAAAAEAVKAAAG
MSTRG.22634.1    -VANVWFA-------RTGPAWGRRAS----------------------YSGLRQTQSTIG
                                ##########                                   


                 
                 ========
Ec-01_005570     EESGESHH
MSTRG.22634.1    SLSSLTQ-
```

```
Parameters used
Minimum Number Of Sequences For A Conserved Position: 2
Minimum Number Of Sequences For A Flanking Position: 2
Maximum Number Of Contiguous Nonconserved Positions: 8
Minimum Length Of A Block: 10
Allowed Gap Positions: None
Use Similarity Matrices: Yes
```

```
Flank positions of the 6 selected block(s)
Flanks: [1  26]  [34  88]  [92  102]  [161  459]  [526  545]  [616  625]  

New number of positions in Ec-01_005570.aa.fasta.trans.aa_ali.fasta-gb:  421  (63% of the original 668 positions)
```
